# A new species of *Euscorpius* Thorell, 1876 (Scorpiones, Euscorpiidae) from south western Turkey

**DOI:** 10.3897/zookeys.348.5943

**Published:** 2013-11-08

**Authors:** Ersen Aydın Yağmur, Gioele Tropea, Fatih Yeşilyurt

**Affiliations:** 1 Alaşehir Vocational School, Celal Bayar University, Manisa, Turkey; 2 Società Romana di Scienze Naturali, Rome, Italy; 3 Kırıkkale University, Science and Art Faculty, Biology Department, Kırıkkale, Turkey

**Keywords:** Scorpion, *Euscorpius*, new species, Turkey

## Abstract

A new scorpion species, *Euscorpius lycius* **sp. n.**, is described based on specimens collected from Muğla and Antalya Provinces, in southwestern Turkey. It is characterized by a standard trichobothrial pattern (*Pv*=8/9, *et*=6, *em*=4, *eb*=4), small size and light brown/reddish coloration. With the description of *Euscorpius lycius* **sp. n.**, the number of valid species of the genus *Euscorpius* in Turkey increases to 5.

## Introduction

The genus *Euscorpius* Thorell, 1876 is one of the most studied taxa of scorpions; however, because of its complexity, its taxonomy changes continuously and is not completely clear. The *Euscorpius* populations of Turkey have been poorly studied, but in the last years several studies are delineating the diversification and distribution of the various forms of this genus (e.g. Fet et al. 2003b; Karataş 2006; Tropea et al. 2012; Yağmur and Tropea 2013). At present only four valid species are recognized in Turkey (not including the new species): *Euscorpius italicus* (Herbst, 1800), *Euscorpius mingrelicus* (Kessler, 1874), *Euscorpius avcii* Tropea et al., 2012 and *Euscorpius rahsenae* Yağmur & Tropea, 2013. *Euscorpius mingrelicus*, which is a species complex, has six described subspecies in Turkey [*Euscorpius mingrelicus mingrelicus* (Kessler, 1874), *Euscorpius mingrelicus ciliciensis* Birula, 1898, *Euscorpius mingrelicus phrygius* Bonacina, 1980, *Euscorpius mingrelicus ollivieri* Lacroix, 1995, *Euscorpius mingrelicus legrandi* Lacroix, 1995, and *Euscorpius mingrelicus uludagensis* Lacroix, 1995)] that need clarification.

Presence of the subgenus *Euscorpius* in Turkey have been reported many times under the name of *Euscorpius carpathicus* or *Euscorpius carpathicus* “complex”, from İstanbul (Hadži 1930; Vachon 1951); Havza (Samsun) (Schenkel 1947); Sinop (Tolunay 1959); Amasya, the Middle Taurus, Borçka (Artvin), Çanakkale, Trakya and Efes (İzmir) (Kinzelbach 1975, 1982); Alanya (Antalya), Bursa Town and Gemlik (Bursa), Ayvacık and Çan (Çanakkale), Sarıyer, Üsküdar and Büyükada Island (İstanbul), Urla (İzmir), Fethiye (Muğla), Sinop Town and Ada vicinity (Sinop) (Karataş 2006); and Dilek Peninsula (Aydın) (Koç and Yağmur 2007). Furthermore, Kinzelbach (1975) recorded *Euscorpius mesotrichus* from Şile (İstanbul) and Prinkipos Island (Büyükada Island) in the Marmara Sea. Further studies (Di Caporiacco 1950; Fet 1997; Fet and Braunwalder 2000; Gantenbein et al. 2001; Fet and Soleglad 2002; Fet et al. 2003a; Tropea et al. 2012; Tropea and Rossi 2011–2012) reported that *Euscorpius mesotrichus* is not an available name, and populations within Kinzelbach’s interpretation, referred to other species such as *Euscorpius tergestinus*, *Euscorpius balearicus*, *Euscorpius sicanus* and other forms.

The new species described herein, *Euscorpius lycius* sp. n., is the third species recognizedin Turkey which “falls” in the subgenus *Euscorpius* as it is understood until now; however, in the present study we do not assign a subgeneric level, since that the subgenus *Euscorpius* currently needs depth studies and new dichotomous keys as has been shown in Tropea (2013).

## Materials and methods

A total of 26 specimens belonging to the new species were collected from Antalya and Muğla Province, in the south-west of Turkey (Fig. 8). Most of specimens were collected in night time from under pine forest while they were siting on the rocks, cracks and garden walls. Some specimens were collected from under stones in pine forests in day time. Comparison material: *Euscorpius avcii*, holotype ♂, Dilek Peninsula National Park, Canyon, Dilek Peninsula, near Davutlar Town, Kuşadası, Aydın, Turkey, 07.10.2005, leg. H. Koç (MTAS); paratypes, 1 ♂, 5 ♀♀, Dilek Peninsula National Park, Canyon, Dilek Peninsula, near Davutlar Town, Kuşadası District, Aydın Province, Turkey, 07.10.2005, leg. H. Koç (MZUF); same data, 1 ♂, 2 ♀♀ (GTC); Abbreviations: *V*: trichobothria on ventral pedipalp chela manus; *Pv*: trichobothria on patella ventral surface; *Pe*: trichobothria on the pedipalp patella external surface; *et*: external terminal; *est*: external subterminal; *em*: external medium; *esb*: external suprabasal; *eb_a_*: external basal *a*; *eb*: external basal; DPS: dorsal patellar spur; DD: distal denticle; MD: median denticles; OD: outer denticles; ID: inner denticles; IAD: inner accessory denticles. Material examined is deposited in the following collections: MZUF: Museo Zoologico ‘La Specola’ dell’Università di Firenze, Florence, Italy; GTC: private collection of Gioele Tropea, Rome, Italy; MTAS: Museum of the Turkish Arachnological Society; MSNB: Museo Civico di Scienze Naturali “E. Caffi”, Bergamo, Italy; ZMSU: Zoology Museum of Sinop University, Turkey; KUAM: Arachnological Museum of Kırıkkale University, Turkey; AZM: Zoology Museum of Alaşehir Vocational School, Celal Bayar University, Manisa, Turkey.

The trichobothrial notations follow Vachon (1974). The morphological measurements are given in millimeters (mm) following Sissom et al. (1990). The morphological nomenclature follows Stahnke (1970), Hjelle (1990), and Sissom (1990); the chela carinae and denticle configuration follows Soleglad and Sissom (2001) and sternum terminology follows Soleglad and Fet (2003); description and terminology of hemispermatophore follows Soleglad and Sissom (2001) and Fet and Soleglad (2002).

## Taxonomy

### Family Euscorpiidae Laurie, 1896
Genus *Euscorpius* Thorell, 1876

#### Euscorpius lycius sp. n.

http://zoobank.org/2146A862-414B-4EB7-9FEE-B5CF2B973395

http://species-id.net/wiki/Euscorpius_lycius

Figs 1 –7

##### Type material.

**Holotype: 1)** 1♂, Turkey, Muğla Province, Fethiye District, Faralya Village, 30.05.2012, 36°29'37"N, 29°08'07"E, 349 m, leg. F. Yeşilyurt & E. A. Yağmur (AZM).

Paratypes: 1) 3♀♀, 4♂♂. Muğla Province, Fethiye District, Faralya Village, 30.05.2012, 36°29'37"N, 29°08'07"E, 349 m, leg. F. Yeşilyurt & E. A. Yağmur (KUAM). Same data but 1♀, 1♂ (AZM) 2♀, 2♂ (GTC) 1♀, 1♂ (MSNB).

2) 1♂. Muğla Province, Fethiye District, Babadağ Mountain, 26.06.2013, 36°28'58"N, 29°12'04" E, 1132 m, leg E. A. Yağmur, M. Kesdek & Y. İlemin (AZM).

3) 1♂. Antalya Province, Kaş District, Gömücü Village, 15.05.2012, 36°24'15"N, 29°42'01"E, 976 m, leg. R. Kaya & A. Akkaya (AZM).

4) 2 juv. Antalya Province, Kaş District, İkizce Village, 5 km North, 13.04.2012, 36°21'30"N, 29°29'00"E, 1140 m, leg. E. A. Yağmur & D. Türk (AZM). Same data but 02.06.2012, 5♀♀, 1♂ (AZM), leg. E. A. Yağmur, M. Örgel & D. Türk (Fig. 8).

**Table 1.** Measurements (in mm) of male holotype and female paratype of *Euscorpius lycius* sp. n.

		Holotype ♂	Paratype ♀
**Total**	Length	21.14	20.91
**Carapace**	Length	3.06	3.48
	Posterior width	3.12	3.42
**Metasoma**	Length	7.94	7.17
**Segment I**	Length	0.98	0.96
	Width	1.08	1.08
**Segment II**	Length	1.20	1.17
	Width	0.91	0.95
**Segment III**	Length	1.36	1.32
	Width	0.87	0.87
**Segment IV**	Length	1.63	1.26
	Width	0.84	0.83
**Segment V**	Length	2.76	2.46
	Width	0.84	0.83
**Telson**	Length	3.24	2.64
**Vesicle**	Length	2.52	1.86
	Width	1.20	0.90
	Height	1.26	0.84
**Aculeus**	Length	0.72	0.78
**Femur**	Length	2.61	2.82
	Width	0.95	1.08
**Patella**	Length	2.58	2.82
	Width	1.02	1.23
**Chela**	Length	5.10	5.58
	Width	1.86	1.98
**Movable finger**	Length	2.70	3

##### Etymology.

The specific epithet refers to the ancient Latin name of the collection area, which is Lycia.

##### Diagnosis.

A small *Euscorpius* species, total length 21–25 mm. Color of adults light brown-reddish with carapace and pedipalps darker. The number of trichobothria on the pedipalp manus ventral surface is 4 (3 *V*+*Et* 1); the number of trichobothria on the pedipalp patella ventral surface usually is 9 (in 86.54% of examined pedipalps); the number of trichobothria on pedipalp patella external surface usually is: *eb*=4, *eb_a_*=4, *esb*=2, *em*=4, *est*=4, *et*=6–7 (*et*=6 in 53.84% and *et*=7 in 36.54% of examined pedipalps). The pectinal teeth count usually is 8 (8 in 66.66% and 9 in 29.16% of examined pectines) in males and 7 in females. Dorsal carinae of the metasomal segments I–IV granulated. Chela with a notch on fixed finger and scalloping of the movable finger in adult males, obsolete in females. Trichobothrium *et* occurs distally to the notch on the fixed finger, *est* occurs above the notch on the fixed finger and *dsb* occurs proximally to the notch of the fix finger.

##### Description of the holotype male.

***Coloration*:** Very light brown-reddish with carapace and pedipalps darker. Granules on the femora of the legs, especially ventrally, dark. The sternites, pectines and genital operculum are very light brownish-whitish.

**Figure 1.**
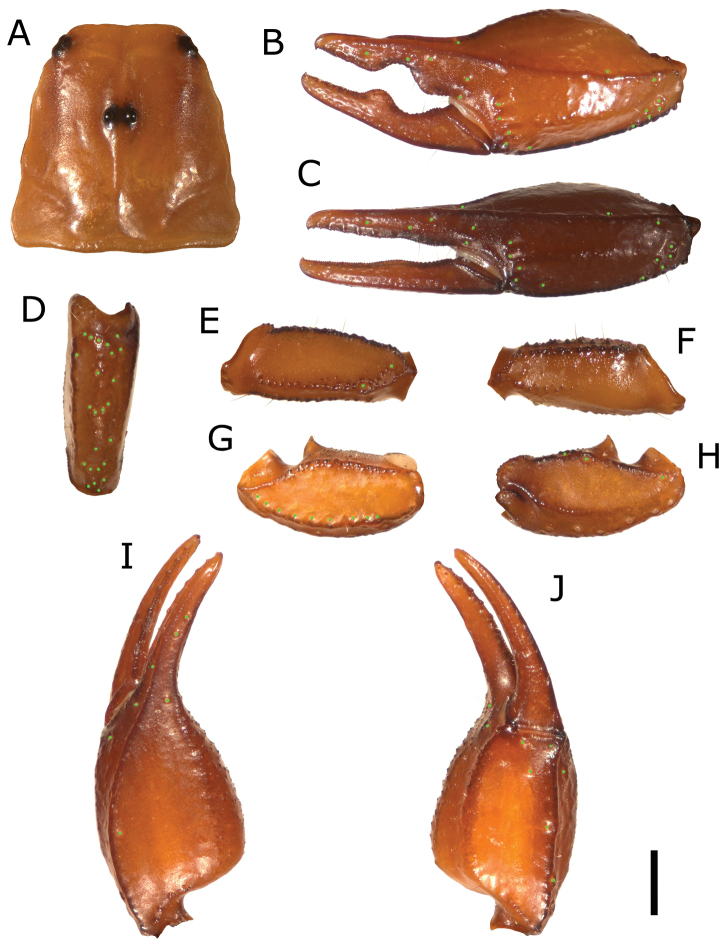
**A** carapace **B** external view of chela of the adult male **C** external view of chela of the adult female **D** external view of pedipalp patella **E** dorsal view of pedipalp femur **F** ventral view of pedipalp femur **G** ventral view of pedipalp patella **H** dorsal view of pedipalp patella **I** dorsal view of chela **J** ventral view of chela. (Scale bar=1 mm).

*Carapace*. Length 3.06 mm; posterior width 3.12. Finely granulated. Distance from the center of the median eyes to the anterior margin of the carapace is 39.21% of the prosoma; the length from the center of the median eyes to the posterior margin of the carapace is 60.79% of the prosoma. Length/Posterior width ratio 0.981 (Fig. 1A).

*Mesosoma*. Tergites finely granulated; sternites smooth. The area of overlap between the sternites is lighter in color. Pectinal teeth count is 9–9. The spiracles are very small and little visible, oval-shaped and it is inclined about 45° downwards towards outside.

**Figure 2.**
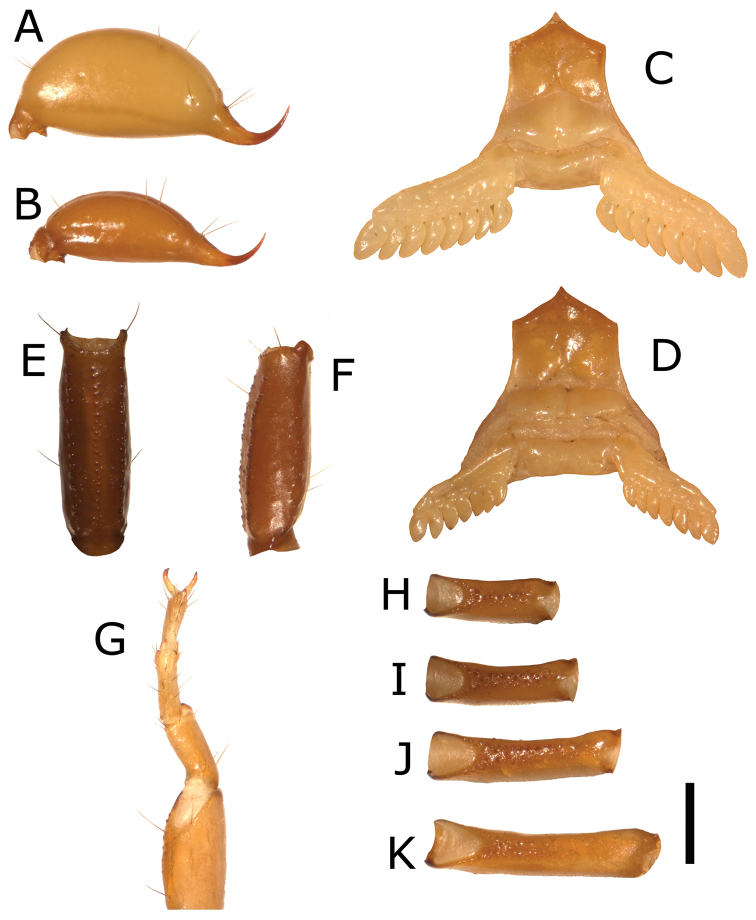
**A** telson of adult male **B** telson of adult female **C** sternopectinal area of adult male **D** sternopectinal area of adult female **E** ventral view of the metasomal segment V **F** lateral view of the metasomal segment V **G** tarsus and basitarsus **H** leg femur I **I** leg femur II **J** leg femur III **K** leg femur IV. (Scale bar=1 mm).

*Metasoma*. Medium size with respect to body length. Dorsal carinae of segment I-IV are granulated, obsolete on the segment V; ventromedian carinae of segment I-III absent, barely visible angularities on the IV, ventromedian carinae on segment V granulated; ventrolateral carinae of segment I absent, on segments II and III obsolete, on segment IV formed by small spaced granules, on segment V is formed by serrulate granules (Fig. 2E, F).

*Telson*. Vesicle weakly swollen; smooth, with ventral setae of different sizes; telson height 1.26; telson length 3.24; vesicle length 2.52; vesicle width 1.20; L/H ratio of the vesicle 2 (Fig. 2A, B).

*Pectines*. Pectinalteeth count 9-9; middle lamellae count 5-5.

*Genital operculum*. Partially divided with genital papillae protruding.

*Sternum*. Pentagonal shape, type 2. Length similar to width, deep posterior emargination.

*Pedipalp*. Coxa and trochanter granulated. Femur: dorsal internal carinae formed by large dark tubercles;dorsal external carinae formed by slightly serrulated and spaced tubercles; intercarinal spaces uniformly granulated with fine granules; external median carinae serrulate; anterior median carinae formed by conical big dark tubercles (Fig. 1E, F). Patella length 2.58; patella width 1.02; dorsal internal carinae dark and crenulate with few larger tubercles distally; dorsal and ventral external carinae rough; ventral internal carinae dark and tuberculate; dorsal intercarinal tegument very finely granulated; ventral intercarinal tegument almost smooth with a few scattered very small granules; internal intercarinal tegument uniformly finely granulate. Dorsal patellar spur well developed (Fig. 1G, H). Chelal carina *D_1_* isdistinctly strong, a bit darker and from smooth to rough; *D_4_* little marked, roughly smooth with a few very low granules; *V****_1_*** isdistinctly strong, rough and dark; *V_3_* with a few scattered very minuscule granules; external carina rough; intercarinal tegument from smooth to rough except between carinae *D_4_* and *V_3_*, granulate. Movable finger dentition: MD form a straight line of very small denticles closely spaced with a DD on the distal tip; OD formed of 7 denticles on movable finger and 6 denticles on fixed finger, immediately outside of MD, their size increases progressively but the terminal denticle is not very pronounced; ID formed of 7 denticles on movable finger and 6 denticles on fixed finger, spaced from MD, their size increases progressively but the terminal denticle is not very pronounced; IAD on both movable and fixed finger formed of 4 small denticles; L/W ratio of the chela 2.74 (Fig. 1I, J)

*Trichobothria*. Chela trichobothria series *V* standard: *V*=4/4 (3 *V*+*Et* 1); patella ventral (*P*v): 9/9; Patella external (*P*e): *et*=5/6, *est*=4/4, *em*=4/4, *esb*=2/2, *eba*=4/4, *eb*=4/4.

*Legs*. legs with two pedal spurs. Tarsal ventral row with 10-14 spinules (including the ventral distal spinule); 3 flanking pairs of tarsal setae adjacent to the ventral spinules row. Little marked granulation present above leg femora, a bit more marked on III leg; dark conical tubercles on ventral leg femora.

*Chelicerae*. smooth, without marbling, uniformly coloured; typical dentition pattern of *Euscorpius* genus (Soleglad and Sissom 2001).

*Variation*. The variation observed in 26 studied specimens (12 males, 14 females) is as follows (left/right asymmetry not specified). Pectinal teeth in males (n=12): 7/8 (1), 8/8 (6), 8/9 (3), 9/9 (2); 8 in 66.66% and 9 in 29.16%; mean=8.25, SD=0.52. Pectinal teeth in females (n=14): 7/7 (12), 8/7 (2); 7 in 92.28% and 8 in 7.72%; mean=7.07, SD=0.26. Pedipalp patella trichobothria *Pv* (n=26): 9/10 (1), 9/9 (20), 8/9 (4), 8/8 (1); 9 in 86.54 % and 8 in 11.54 %; mean=8.90, SD=0.35. Pedipalp patella trichobothria *Pe* (n=26): *et*=?/6 (1), 5/6 (3), 6/6 (12), 7/7 (9), 7/8 (1); 6 in 54.90 % and 7 in 37.25 %; mean=6.35, SD=0.56. *est*=4/4 (26); *em*=4/3 (3), 4/4 (23); *esb*=2/2 (26); *eb_a_*=4/3 (2), 4/4 (24); *eb*=3/4 (1), 4/4 (25). The telson vesicle in males is more swollen than in females: average L/H ratio of the vesicle is 1.93 in male and 2.28 in females. Dorsal patellar spur well developed. Average value of the length from center median eyes to anterior margin of the carapace is 40.30% of the carapace length. Average value of the length from center median eyes to posterior margin of the carapace is 59.70% of the carapace length.

*Hemispermatophore*. Were checked both right and left hemispermatophore of 5 specimens. Well developed lamina with well developed basal constriction, tapered distally; truncal flexure present and well developed; capsular lobe complex well developed, with acuminate process; ental channel spinose distally, exhibiting 8–11 tines in its crown (Fig. 7). The number of tines of the crown may be different between the right and the left hemispermatophore.

**Figure 3.**
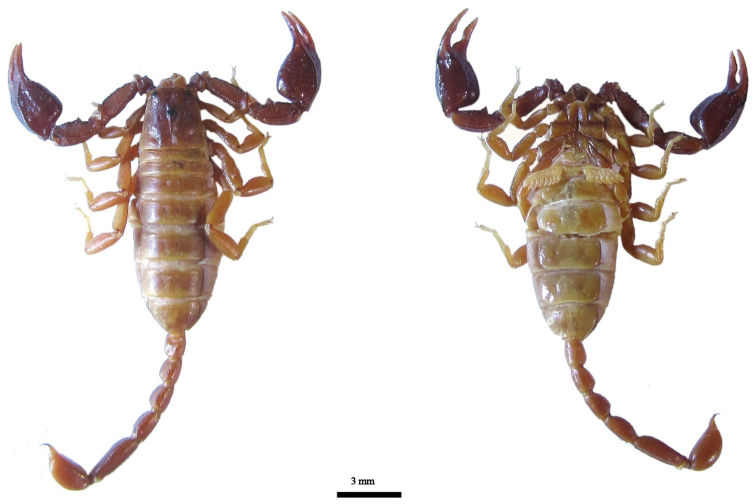
Dorsal and ventral views of *Euscorpius lycius* sp. n. male.

**Figure 4.**
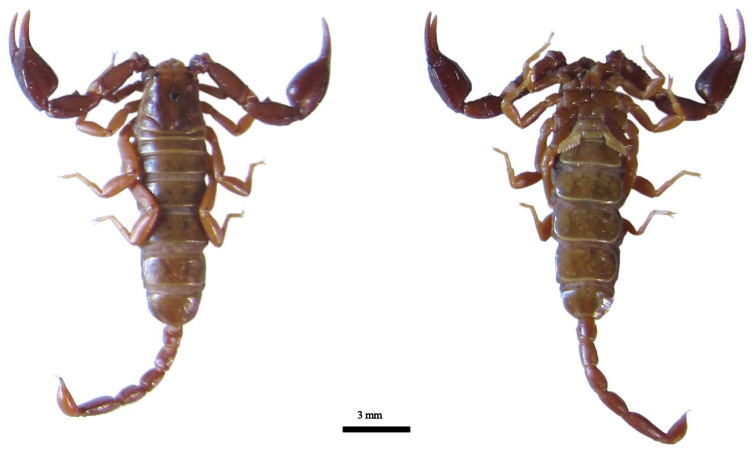
Dorsal and ventral views of *Euscorpius lycius* sp. n. female.

**Figure 5.**
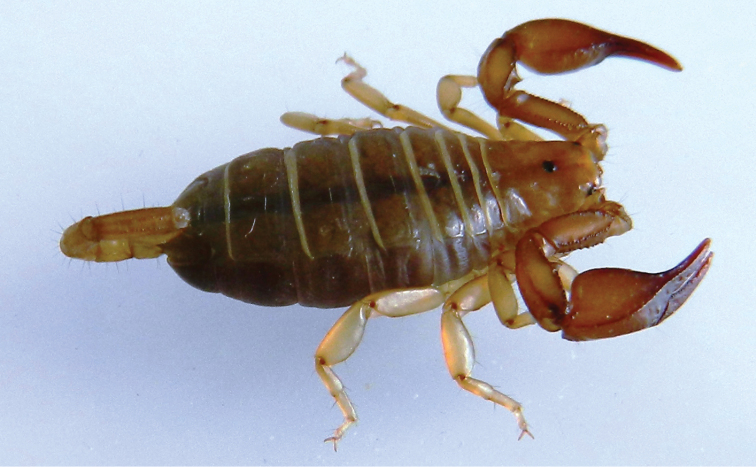
An alive female specimen of *Euscorpius lycius* sp. n.

**Figure 6.**
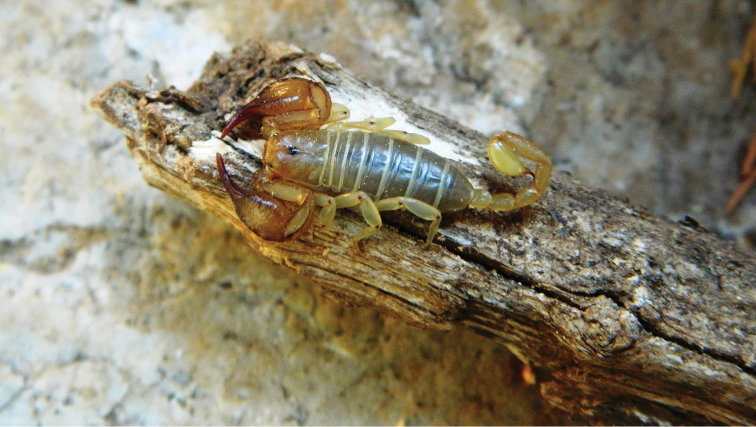
A male *Euscorpius lycius* sp. n. in its natural habitat.

**Figure 7.**
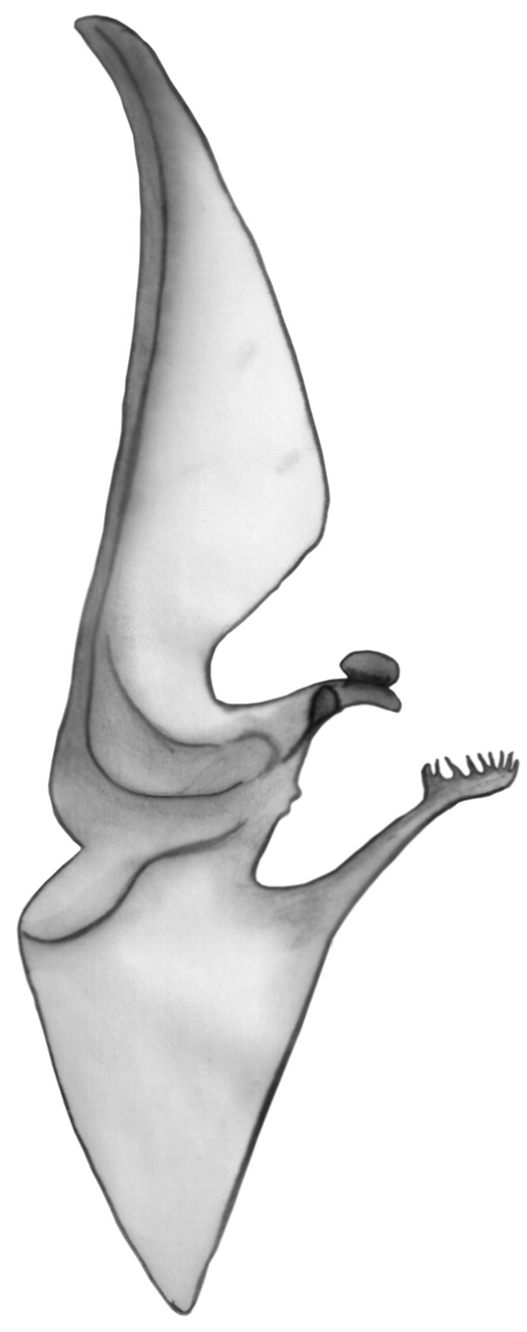
Left hemispermatophore of *Euscorpius lycius* sp. n.

**Figure 8.**
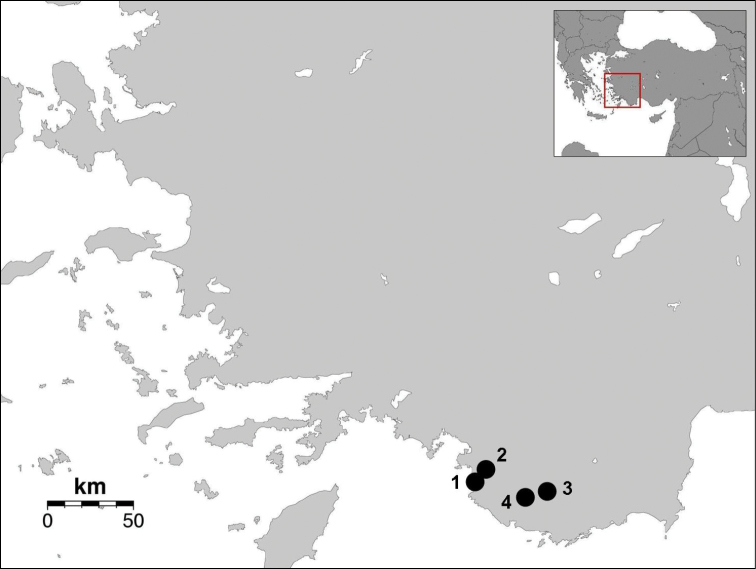
Sampling map of *Euscorpius lycius* sp. n. **1** Muğla, Faralya Village **2** Muğla, Babadağ Mountain **3** Antalya, Gömücü Village **4** Antalya, İkizce Village.

**Table 2.** Pectinal tooth and trichobothrial counts of *Euscorpius* species discussed in this paper. Between the brackets are the values most found.

Species	*Dp* ♂	*Dp* ♀	*Pv*	*Pe*-*et*	*Pe*-*est*	*Pe*-*em*	*Pe*-*esb*	*Pe*-*eba*	*Pe*-*eb*
*Euscorpius lycius* sp. n.	8	7	9	6–7	4	4	2	4	4
*Euscorpius avcii*	8	7	7	5–6 (5)	4	4	2	4	4
*Euscorpius rahsenae*	9	7	8	5–6 (6)	4	4	2	4	4
*Euscorpius candiota*	8–9	7	9-10	6–7	4	4	2	4	4
*Euscorpius ossae*	9	7	7–8 (7)	5–6 (5)	4	4	2	4	4
*Euscorpius koschewnikowi*	8	6–7	8	5–6	4	4	2	4	4
*Euscorpius scaber*	10–11	8	8	5–6 (6)	4	4	2	4	4
*Euscorpius carpathicus aegaeus*	9	8	8	6	4	4	2	4	4

**Table 3.** Pectinal tooth and trichobothrial serie *Pv* and *Pe*-*et* counts of *Euscorpius* species discussed in this paper: max-min (average) {number of pectines and pedipalps examined}.

Species	*Dp* ♂	*Dp* ♀	*Pv*	*Pe*-*et*
*Euscorpius lycius* sp. n.	7–9 (8.25) {24}	7–8 (7.07) {28}	8–10 (8.90) {52}	5–7 (6.35) {51}
*Euscorpius avcii*	7–9 (8.07) {58}	6–7 (6.79) {100}	6–8 (7.04) {158}	5–6 (5.36) {158}
*Euscorpius rahsenae*	8–10 (8.91) {36}	6–9 (7.20) {82}	7–9 (7.89) {118}	5–6 (5.78) {118}
*Euscorpius candiota*	8–9 (8.60) {16}	5–8 (6.87) {28}	8–10 (9.44) {46}	5–7 (6.52) {46}
*Euscorpius ossae*	8–10 (9.07) {14}	6–8 (7.25) {40}	6–9 (7.29) {55}	4–6 (5.36) {55}
*Euscorpius scaber*	9–13 (10.53) {53}	6–10 (7.85) {212}	7–10 (7.96) {273}	4–8 (5.86) {257}
*Euscorpius carpathicus aegaeus*	9–10 (9.16) {6}	8 (8.00) {4}	7–8 (7.9) {10}	5–6 (5.9) {10}

### Discussion and comparison

Karataş (2006) reported two populations of the subgenus *Euscorpius* from Turkey as “*Euscorpius* sp.1” and “*Euscorpius* sp.2”. The first population has been reported from Bursa, Çanakkale, İstanbul, İzmir, and Sinop Provinces; the second has been reported from Antalya and Muğla Provinces. *Euscorpius lycius* sp. n. occurs within the area of the second population (south-west). Karataş (2006) reported that in both “*Euscorpius* sp.1” and “*Euscorpius* sp.2”, *V4* was situated on the *ventral* surface, internally from the external ventral carina, but *Euscorpius lycius* sp. n. specimens, as well as those from İstanbul that coincide with “*Euscorpius* sp.1” of Karataş (2006), have the trichobothrium *V4* situated on the external surface. It is probable that Karataş (2006) misinterpreted the trichobothrial nomenclature of the chela.

*Euscorpius lycius* sp. n. is “related” to the subgenus *Euscorpius* as it is understood until now, thus clearly distinguished from *Euscorpius italicus* and *Euscorpius mingrelicus*. However, in the present study we do not assign the subgeneric level, since that the subgenus *Euscorpius* currently needs depth studies and new dichotomous keys as has been shown in Tropea (2013). The only valid species “related” to the subgenus *Euscorpius* in Turkey are *Euscorpius avcii* and *Euscorpius rahsenae*.

*Euscorpius avcii* was described from Dilek Peninsula as an oligotrichous, small *Euscorpius*, with a length of 24–28 mm, light brown to brown-reddish colored with the carapace and pedipalps darker, and legs and telson lighter (Tropea et al. 2012). These two species are similar in colour and size, although *Euscorpius lycius* sp. n. is on average smaller. However, they may be differentiated as follows: (1) *Pv* count is usually 7 in *Euscorpius avcii* and 9 in *Euscorpius lycius* sp. n.; 2) *Pe*-*et* series is generally 5 in *Euscorpius avcii* and 6 in *Euscorpius lycius* sp. n.; (3) *Euscorpius avcii* has the metasomal segments almost smooth while *Euscorpius lycius* sp. n. exhibits noticeable granulated carinae; (4) dorsal patellar spur weakly developed in *Euscorpius avcii*, but well developed in *Euscorpius lycius* sp. n.

*Euscorpius rahsenae* was described from Marmara Region as a medium sized *Euscorpius*, total length 27–34 mm,color very light brown-yellowish with carapace and pedipalps a little darker, legs, telson and chelicerae lighter (Yağmur and Tropea 2013). It is possible to differentiate this species from *Euscorpius lycius* sp. n. as follows: (1) *Pv* count is usually 8 in *Euscorpius rahsenae* and 9 in *Euscorpius lycius* sp. n.; (2) *Euscorpius lycius* sp. n. has the trichobothria *et-est/est-dsb* on fixed finger more proximal of *Euscorpius rahsenae*, in fact in *Euscorpius lycius* the trichobothrium *et* occurs distally to the notch on the fixed finger, *est* occurs above the notch on the fixed finger and *dsb* occurs proximally to the notch of the fix finger (similar to *Euscorpius avcii*), while in *Euscorpius rahsenaeet* and *est* occur distally to the notch on the fixed finger and *dsb* occurs above the notch of the fixed finger; (3) *Euscorpius lycius* sp. n. is on average smaller than *Euscorpius rahsenae* sp. n. (21–25 mm and 27–34 mm, respectively).

Below, we compare *Euscorpius lycius* sp. n. with some other forms present in the Aegean area: *Euscorpius sicanus* (C. L. Koch, 1837) complex; *Euscorpius koschewnikowi* Birula, 1900; *Euscorpius candiota* Birula, 1903; *Euscorpius scaber* Birula, 1900; *Euscorpius ossae* Di Caporiacco, 1950; and *Euscorpius carpathicus aegaeus* Di Caporiacco, 1950.

*Euscorpius sicanus* complex is widespread in mainland Greece and some Aegean islands (Fet et al. 2003a; Tropea and Rossi 2012), and can be easily distinguished from *Euscorpius rahsenae* sp. n. by the trichobothrial *eb* series, 5 in *Euscorpius sicanus* complex and 4 in *Euscorpius lycius* sp. n.

*Euscorpius koschewnikowi* is a medium to large sized species (up to 46 mm), medium to dark brown in color, slender appearance with well developed dorsal patellar spur and all metasoma segments longer than wide. In addition, according to Fet and Soleglad (2002) the exceptionally slender and smooth metasoma are key diagnostic characters of this species. *Euscorpius lycius* sp. n. mainly differs from *Euscorpius koschewnikowi* with a significantly smaller average size; its metasomal segments are not smooth, and the first segment not longer than wide.

*Euscorpius candiota* differs from *Euscorpius lycius* sp. n. for: (1) the metasomal carinae on segments II–IV smooth to obsolete (Fet et al. 2013) while *Euscorpius lycius* sp. n. has the dorsal carinae of the segment I–IV granulated; (2) *Euscorpius candiota* is larger in size, about 40 mm (Fet et al. 2013) versus 21–25 mm in *Euscorpius lycius* sp. n. Furthermore *Euscorpius candiota* tends has a higher *Pv*, *Pe*-*et* and pectinal teeth count in males and it is endemic of Crete island.

*Euscorpius scaber* is a scorpion from the northern Aegean area, it is distinguished from *Euscorpius lycius* sp. n. by (1) a higher number of pectinal teeth, *Dp* 10/11 in males and 8 in females (Fet et al. 2013) versus 8 in males and 7 in females in *Euscorpius lycius* sp. n.; (2) *Euscorpius scaber* has a *Pv*=8 versus 9 in *Euscorpius lycius* sp. n.; (3) *Euscorpius scaber* is heavily granulated (Fet 1985; Tropea et al. 2012; Yağmur and Tropea 2013, Fet et al. 2013), as the name suggests and darker, whereas *Euscorpius lycius* sp. n. is light brownish-reddish, without particularly accentuated granulation. In addition *Euscorpius scaber* occurs in north-east of Greece.

*Euscorpius ossae* is an oligotrichous species, dark brown in colour with lighter legs and telson. It was described from Mount Ossa, in Thessaly. This form can be distinguished from *Euscorpius lycius* sp. n. mainly by *Pv*=7 and *et*=5, compared to *Pv*=9 and *Pe*-*et*=6–7 (generally 6) and its dark colour.

*Euscorpius carpathicus aegaeus* is a light colored form described from the island of Antiparos, in the central-southern part of the Aegean Sea. It is probably endemic in few islands in the central-south Aegean Sea. In addition, it is described as uniformly light yellow in colour and females with a pectinal teeth count of 8 with metasomal segments almost smooth (Di Caporiacco 1950), while *Euscorpius lycius* sp. n. is light brownish-reddish, pectinal teeth count 7 in females and metasomal segments with granulated carinae.

### Ecology

The specimens of *Euscorpius lycius* sp. n. were collected between 349 and 1140 m a.s.l. Most of the specimens were collected in night time in the pine forest while they were siting on the rocks, cracks and garden walls. The remaining specimens were collected in day time from under stones in pine forests.

*Euscorpius lycius* sp. n. specimens from Faralya Village were collected on wall stones and garden walls humid in shady places with a lot of stones covered by moss (Fig. 10). This place is at the top of high rocky wall and very close to sea. Other specimens were collected from red pine (*Pinus brutia* Ten.) and Lebanon cedar (*Cedrus libani* A. Rich.) forest that also includes kermes oak bushes (*Quercus coccifera* L.), in Gömücü Village and İkizce Village (Antalya Province). These two areas have high elevation, are always cool and are located about ten kilometers from the sea. Babadağ Mountain (Muğla Province) locality has same ecological features with these localities. Therefore these three localities are always humid, they include stones covered by moss.

**Figure 9.**
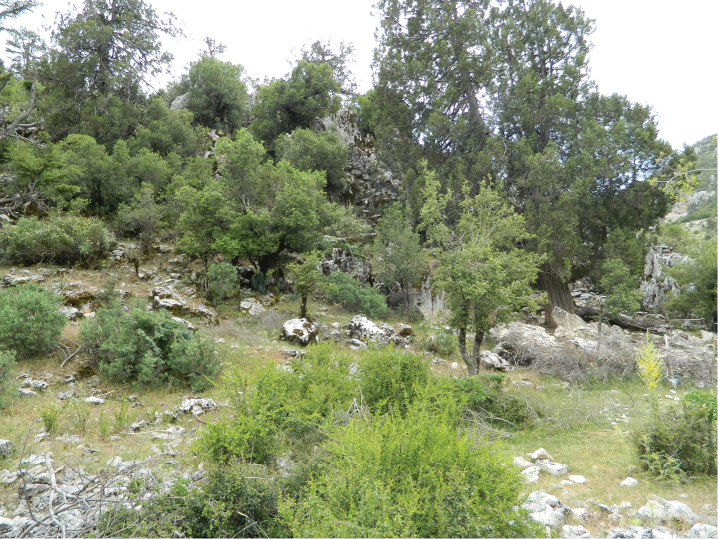
The forest habitat in İkizce Village.

All localities are humid and cool, with calcareous stones covered with moss, where the specimens of *Euscorpius lycius* sp. n. were usually found. We observed that specimens prefer cracks of mossy rocks (Fig. 9). We accept the presence of moss as an indicator during our field trips. Areas with moss are potential places where to find specimens of *Euscorpius*.

**Figure 10.**
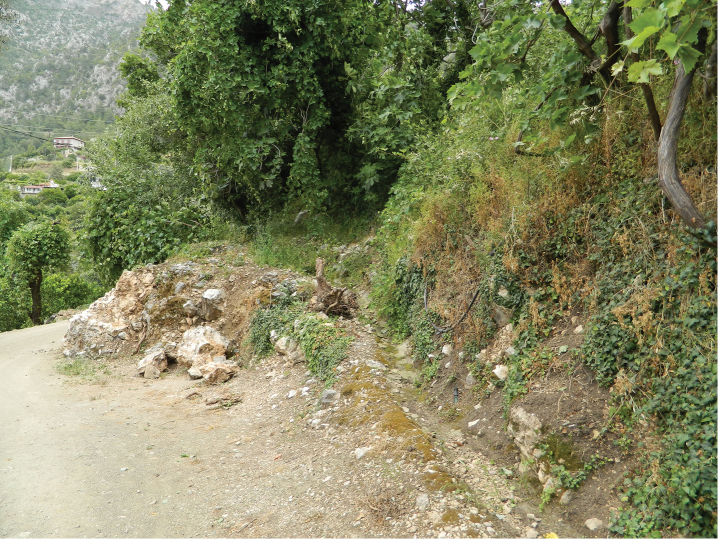
Habitat in Faralya Village.

## Conclusion

Taxonomy of *Euscorpius* genus is complicated and still unresolved throughout its range, because of type specimens lost, lack of specimens from many areas and existence of cryptic species complex, which exhibiting the same, or very similar, standard characters.

The species described herein, *Euscorpius lycius* sp. n., is one of those forms of *Euscorpius* with standard characters shared by several species. This condition of cryptic species complex, is known throughout the range of the genus *Euscorpius*, however it is much more expressed in the band that includes Greece and western Turkey. Additional morphological features that simplify the division between the species of the genus *Euscorpius* should be found, but at the moment the only way to identify the various species is to combine a set of characters, primary and secondary, the area of origin and a certain number of specimens available.

Further studies are in progress to understand the quantity and distribution of the different species and populations of the genus *Euscorpius* in Turkey and their relationship with the Greek populations.

With the description of *Euscorpius lycius* sp. n., the number of valid species of the genus *Euscorpius* in Turkey increases to 5.

## Supplementary Material

XML Treatment for Euscorpius lycius
